# Comparative analyses of six solanaceous transcriptomes reveal a high degree of sequence conservation and species-specific transcripts

**DOI:** 10.1186/1471-2164-6-124

**Published:** 2005-09-14

**Authors:** Willem Albert Rensink, Yuandan Lee, Jia Liu, Stacy Iobst, Shu Ouyang, C Robin Buell

**Affiliations:** 1 The Institute for Genomic Research, 9712 Medical Center Dr., Rockville MD, 20850, USA

## Abstract

**Background:**

The Solanaceae is a family of closely related species with diverse phenotypes that have been exploited for agronomic purposes. Previous studies involving a small number of genes suggested sequence conservation across the Solanaceae. The availability of large collections of Expressed Sequence Tags (ESTs) for the Solanaceae now provides the opportunity to assess sequence conservation and divergence on a genomic scale.

**Results:**

All available ESTs and Expressed Transcripts (ETs), 449,224 sequences for six Solanaceae species (potato, tomato, pepper, petunia, tobacco and *Nicotiana benthamiana*), were clustered and assembled into gene indices. Examination of gene ontologies revealed that the transcripts within the gene indices encode a similar suite of biological processes. Although the ESTs and ETs were derived from a variety of tissues, 55–81% of the sequences had significant similarity at the nucleotide level with sequences among the six species. Putative orthologs could be identified for 28–58% of the sequences. This high degree of sequence conservation was supported by expression profiling using heterologous hybridizations to potato cDNA arrays that showed similar expression patterns in mature leaves for all six solanaceous species. 16–19% of the transcripts within the six Solanaceae gene indices did not have matches among Solanaceae, Arabidopsis, rice or 21 other plant gene indices.

**Conclusion:**

Results from this genome scale analysis confirmed a high level of sequence conservation at the nucleotide level of the coding sequence among Solanaceae. Additionally, the results indicated that part of the Solanaceae transcriptome is likely to be unique for each species.

## Background

The Solanaceae family encompasses a number of species of agronomic and ornamental importance. With regards to cultivation for food consumption, in 2003, potato was the world's fifth largest crop in world-wide production acreage and the solanaceous vegetables tomato, eggplant, and pepper ranked 11th, 19th, and 22nd, respectively [1]. Species grown for ornamental purposes include petunia and *Nicotiana *species. While not consumed for food, these horticultural species are a substantial component of the US agronomic economy. For example, petunia represents greater than $148M output per year in the US [2]. Tobacco represents another crop of significant economical importance with $1.6B in crop value in 2003 [3]. A close relative of tobacco, *Nicotiana benthamiana*, has been utilized as an experimental model for viral research and disease resistance studies. Coupled with the robust ability of virus induced gene silencing to silence transcripts [4], *N. benthamiana *has emerged as a model species for disease resistance research.

The Solanaceae have been bred and developed for a variety of purposes. Potato has been bred for tubers (modified stems) while tomato, pepper, and eggplant have been bred for enhanced fruit production. Likewise, petunia has been bred and selected for floral phenotypes while tobacco has been bred for leaf size. While these modern varieties are accentuated for particular morphological features, these species share common taxonomic features of the Solanaceae such as alternate leaves, flower parts in five, and fruit as a berry or capsule. Compared with other plant families such as the Poaceae, the range of genome sizes of solanaceous species is fairly narrow, ranging from 900 to 4600 Mb per haploid genome [5]. Early studies of the Solanaceae genome revealed conservation of gene content among potato, tomato, tobacco, petunia, and eggplant. These studies employed relatively small scale cross-hybridization studies using cDNA and random genomic DNA clones [6] in which a set of 20 tomato cDNA clones were hybridized with a panel of solanceous species including *Lycopersicon*, *Solanum*, *Datura*, *Petunia*, and *Nicotiana*. For the cDNA clones, there was strong hybridization across the Solanaceae; however, with the genomic clones (50 in total), there was a reduced degree of cross-hybridization with the non-*Lycopersicon *species. These data suggested conservation among the coding sequences while the non-coding sequences had undergone substantial divergence.

Conserved gene content prompts the question of conserved gene order, i.e. synteny across the Solanaceae. A number of solanaceous species have a base chromosome number of 12 including the main vegetable crop species potato, tomato, pepper and eggplant. Using markers developed from tomato, a strong degree of co-linearity between potato and tomato has been demonstrated with the differences attributable to paracentric inversions occurring between these two species [7,8]. Using the same approach in pepper, 18 homologous linkage blocks between tomato and pepper could be identified [9]. In eggplant, tomato markers yet again revealed syntenic regions among tomato and eggplant [10]. While these synteny studies utilized anonymous DNA clones as markers, comparative mapping of phenotypes such as fruit morphology [11], pigmentation [12] and disease resistance [13] revealed syntenous mapping of these traits across the Solanaceae.

These early studies relied heavily on cDNA and random genomic clones. The advent of high throughput sequencing projects such as Expressed Sequence Tags (ESTs) [14] has resulted in the generation of hundreds of thousands of sequences for solanaeous species. For this study, a total of 441,154 ESTs were collected from the public database (dbEST) representing the solanaceous species tomato (162,621), potato (189,864), pepper (29,894), tobacco (26,497), and *N. benthamiana *(26,918). The available solanaceous ESTs, along with Expressed Transcripts (ETs), available in Genbank, can be clustered into gene indices [15] that represent a non-redundant set of transcripts and facilitate analysis of redundant EST collections. Using potato and tomato gene indices, a comparative analysis of tomato and potato ESTs revealed that approximately 80% of the potato ESTs had a significant sequence match with a tomato EST at the nucleotide level (E value cutoff of 10^-10^) [16].

In this study, we report the construction and comparative analyses of gene indices for six solanaceous species (tomato, potato, tobacco, pepper, petunia and *N. benthamiana*). These gene indices represent a total of 116,207 non-redundant sequences which we have utilized to assess sequence conservation among the Solanaceae on a genomic scale. We significantly extended previous studies on sequence similarity and conservation among these species as well as documented more thoroughly the characteristics of the coding portion of the Solanaceae genome. Using computational methods, we have identified putative orthologs among these species and generated a phylogenetic tree to ascertain the relationship and sequence divergence among these species. In addition to these computational approaches, we assessed the similarity of expression profiles in mature leaves to experimentally validate the sequence conservation of these species using heterologous hybridization to potato cDNA microarrays. The comparison of the solanaceous transcripts to the predicted proteomes of the near-complete genome sequences of Arabidopsis, rice, as well as to 21 other plant gene indices resulted in the identification of solanaceous transcripts without putative homologs, suggesting that a portion of these transcripts have a high likelihood of being unique to the Solanaceae. These analyses provide insight into the overall sequence conservation among eudicots (Arabidopsis and Solanaceae) as well as between the Solanaceae and the monocots (i.e., rice).

## Results

### Assembly of sequences into gene indices for potato, tomato, petunia, tobacco, pepper, and *N. benthamina*

A total of 446,248 sequences for six different Solanaceae family members were retrieved from Genbank, including dbEST. All sequences were derived from multiple libraries. The differences in relative expression levels of the various transcripts will result in a large number of redundant transcripts within these libraries. In order to analyze the transcriptome on the single transcript level, all sequences for each species were assembled into a gene index resulting in a total of 116,207 unique sequences over all six species. Abundant sequences could be assembled into longer, more accurate consensus transcripts termed tentative consensus (TC) sequences. Less abundant or lowly expressed transcripts could not be assembled into larger contigs resulting in singletons in the assemblies, termed singleton ESTs or singleton ETs. A summary of the composition of each gene index is shown in Table 1. The potato gene index contained the highest number of EST and ET sequences (190,851) and petunia the lowest number (8,690). For these species, the number of singleton sequences remaining after assembly is an indication of the level of sequencing and the diversity of the libraries selected for sequencing. Potato (44%), tomato (48%), and *N. benthamiana *(49%) have the lowest number of singleton sequences indicating a better coverage of the respective transcriptome when compared to the higher number of singletons in tobacco (82%), pepper (67%), and petunia (64%). Additional EST sequencing may reduce the number of singletons as it will allow for collapsing of singletons into contigs with increased coverage and representation of the transcriptome.

**Table 1:** Summary of gene indices of potato, tomato, pepper, tobacco, *Nicotiana benthamiana *and petunia. EST: expressed sequence tag; ET: expressed transcript; TC: tentative consensus; sEST: singleton EST; sET: singleton ET. TCs are the assembled clusters of redundant and overlapping EST and ET sequences. The total unique sequences for each gene index are created by combining the TCs, sETs, and sESTs.

	Total EST	Total ET	TC	sEST	sET	Total Unique Sequences	Average length
Potato	189864	987	21063	17077	99	38239	796
Tomato	162621	1587	16268	15392	178	31838	690
Pepper	29894	259	4172	8788	43	13003	542
*N. benthamiana*	26918	76	3799	3707	48	7554	536
Petunia	8336	354	1416	2883	167	4466	908
Tobacco	26497	1831	3023	17385	699	21107	571

### Assessment of the transcript sampling

The sequences used for the construction of the gene indices were generated from various diverse libraries that cover different treatments and stages of development (Table 2). The tissue sources used for library construction and sequencing largely reflected the various agronomic usages and research foci of the different Solanaceae species. For petunia and tomato, most of the sequences were generated from flower libraries as well as fruit libraries, reflecting the research interests in flower and fruit development for petunia and tomato. In contrast, for potato a large number of sequences were generated from stolons and tubers (Table 2). From all species, sequences from leaves were available, in some instances challenged with various stressors. As described below, 76–78% of the potato and tomato sequences have significant matches with each other although the sources of the libraries were very different, i.e. flower and fruit vs. tuber and stolon. For the *Nicotiana *species, tobacco and *N. benthamiana*, most sequences were generated from mixed tissue or callus libraries. This resulted in higher unique transcript discovery rates as judged from the ratio of the total number of sequences versus the number of unique sequences (Table 1). It should be noted that seed libraries, which may contain additional distinct transcripts, were not used for the sequencing in any of the six species examined in this study.

**Table 2:** Tissue representation of EST sequences among the gene indices. For each species, the origin of the library was determined and the total number of sequences from each source calculated. a. For the potato ESTs, 62,931 of the Mixed/Other ESTs were derived from a series of stolon and tuber cDNA libraries. b. For the *N. benthamiana *ESTs, 18,817 of the Mixed/Other ESTs were derived from a single cDNA library constructed by pooling mRNA from abiotic and biotic stressed leaves, roots, and callus.

	Leaf	Root	Flower	Fruit	Callus/Susp. Culture	Mixed/Other	NA	Total
Potato	26874	15634	5282	0	22040	119735^a^	299	189864
Tomato	20724	15000	45684	31684	22187	27178	164	162621
Pepper	5639	5165	3436	15640	0	0	14	29894
*N. benthamiana*	8099	0	0	0	0	18817^b^	2	26918
Petunia	72	0	6618	1646	0	0	0	8336
Tobacco	561	9	218	0	19578	5924	207	26497

### Analysis of the GC content of Solanaceae gene indices

**Figure 1:**
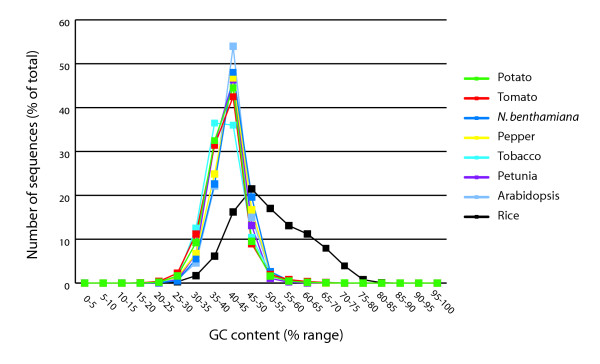
**Analysis of the GC content of the six Solanaceae gene indices, Arabidopsis, and rice. **The average GC content range was calculated for each transcript for the Solanaceae gene indices as well as Arabidopsis and rice.

We analyzed the GC content (ratio of guanine and cytosine) of all of the sequences. It has been shown that Poaceae have GC rich genomes and the transcripts cover a broad range of GC content, whereas eudicot genomes have a lower GC content and transcripts have narrow symmetrical distribution of GC content [17]. The GC content range of the transcripts of the gene indices of the six Solanaceae species was determined (Figure 1). To provide a reference, the GC content range of the Arabidopsis (eudicot) and rice (monocot) gene indices was determined as well. The observed GC content of the Solanaceae gene indices is very similar and in accordance with Arabidopsis. All have a very symmetrical distribution. The average GC content for the majority of transcripts ranges from 40–45%, which is similar to what has been reported previously [18]. The only exception to this distribution were the tobacco transcripts which showed a slightly different profile with an overall lower GC content, in contrast to that previously reported [18] and other Solanaceae species examined in this study.

### Functional annotation of the gene indices

**Figure 2:**
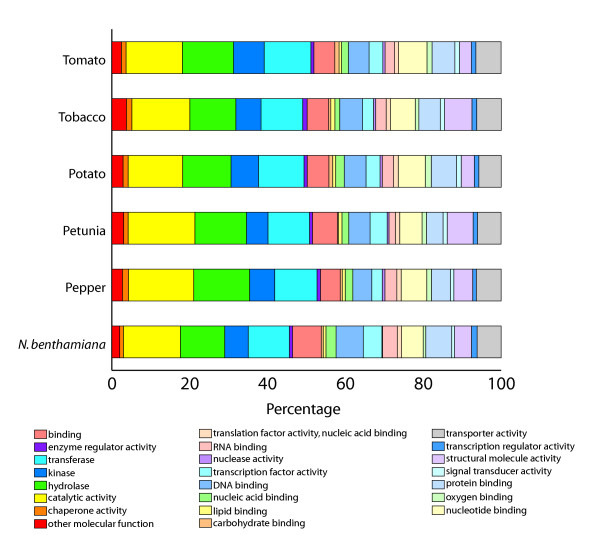
**Assignment of Gene Ontology terms to the Solanaceae gene indices. **Plant GOSlim terms were assigned to the six Solanaceae gene indices in the categories indicated.

Automated annotation of the gene indices was performed as part of the assembly pipeline. In addition, Gene Ontology (GO) terms which provide a more global representation of the gene functions in a controlled vocabulary [19] were assigned to the consensus transcripts of the gene indices. The functional annotations of GO were further reduced using GO-Slim terms, which provide a more accurate GO assignment by assigning a higher level annotation in the GO hierarchy. The GO slim assignments for the six Solanaceae gene indices are shown in Figure 2. A total of 51,830 sequences within the six Solanaceae gene indices were assigned GO-Slim terms. The largest functional categories were catalytic activity (14–17%), hydrolase activity (11–14%) and transferase (11–12%). Overall, the relative composition of the sampled transcriptome over the various functional categories was very similar among the Solanaceae species. In addition, for every species, representative clones could be annotated to every functional GOSlim category, further supporting the representative coverage of the transcriptome throughout all six species. These data indicate that, although the number of sequences and cDNA library sources differ between the six gene indices, the relative functional composition of the transcripts sampled is very similar, further validating the genomic scale comparisons of this study.

### Sequence conservation among six Solanaceae species

**Figure 3:**
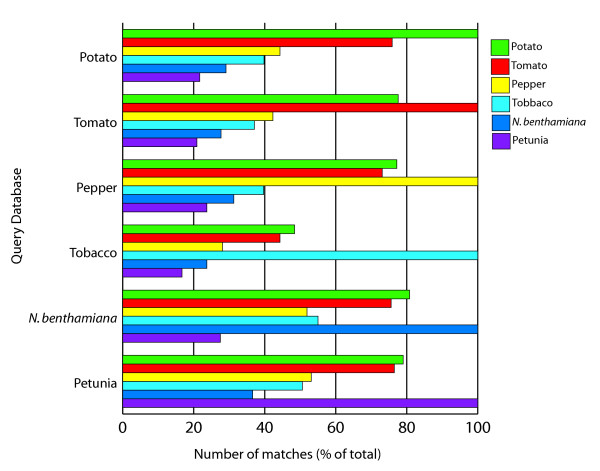
**Percentage of BLASTN matches among Solanaceae gene indices**. Each gene index (query database) was searched against each Solanaceae gene index (color bars). A BLAST score E value cutoff of 10^-10 ^was used for significant sequence matches. Shown is the percentage of transcripts in each Solanaceae gene index.

Previous reports of sequence conservation within the Solanaceae were based on a relatively small number of genes [6]. The availability of six gene indices allowed for the first genomic scale comparisons of sequence similarity between multiple solanaceous species. Pair-wise sequence comparisons of all gene indices were performed using BLASTN [20] and an E value cutoff of 10^-10 ^was used as a minimum cutoff for significant sequence similarity at the nucleotide level. The results are shown in Figure 3. The number of similar sequences between different gene indices is dependent on the number of sequences available, the depth of sequencing from the various libraries, and the tissue diversity represented in the EST collections. For example, comparison of tomato and potato, the largest gene indices, revealed that 76–78% of the sequences had a match in the respective gene index. For the smaller gene indices, such as *N. benthamiana*, 81% of the sequences had matches in potato, whereas the reciprocal comparison revealed only 29% similar sequences which can be attributed to the lower number of sequences present in the *N. benthamiana *gene index. As expected, increasing the stringency (E value 10^-25^) resulted in a lower percentage of matches (data not shown). The similarities at the nucleotide level were paralleled at the protein level as revealed by TBLASTX searches (data not shown).

Sequence comparisons of the Solanaceae species were further refined by the identification of putative orthologs at the nucleotide level among the Solanaceae gene indices. Orthologs are defined as genes with a common ancestor before speciation and which have retained their biological function. The approach we used to identify orthologs [21] utilizes a reciprocal best hit method and was applied to the six Solanaceae gene indices (Table 3). For potato and tomato, the percentage of sequences (39–47%) for which putative orthologs could be identified was lower than the percentage of sequences with significant matches (76–78%), indicating that the identification of orthologs is a more stringent approach for the identification of transcripts with a conserved function. Overall, with the exception of tobacco, 47–60% of the sequences had a reciprocal best match within one of the Solanaceae gene indices and could be classified as a putative ortholog. The clusters of orthologous genes are available in supplemental Table 1 [see Additional file 1].

**Table 3:** Identification of orthologs among solanaceous species. Number and percentages of reciprocal best hit pairs determined by BLAST searches (E value cutoff 10^-10^) were listed and the percentages of the total unique sequences of the species (first column) were calculated.

	Total	*N. benthamiana*	Pepper	Petunia	Potato	Tobacco	Tomato
*N. benthamiana*	7554		2275 (30%)	1050 (14%)	3738 (49%)	2372 (31%)	3520 (47%)
Pepper	13003	2275 (17%)		1688 (13%)	7061 (54%)	3273 (25%)	6762 (52%)
Petunia	4466	1050 (24%)	1688 (38%)		2689 (60%)	1618 (36%)	2591 (58%)
Potato	38239	3738 (10%)	7061 (18%)	2689 (7%)		6337 (17%)	14911 (39%)
Tobacco	21107	4120 (20%)	3273 (16%)	1618 (8%)	6337 (30%)		5869 (28%)
Tomato	31838	3520 (11%)	6762 (21%)	2591 (8%)	14911 (47%)	5859 (18%)	

**Figure 4:**
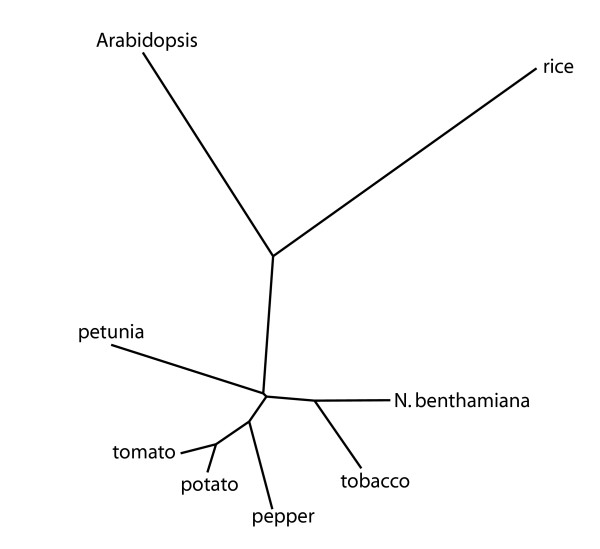
**Sequence divergence among solanaceous species. **Orthologous genes (308) were identified among all eight species indicated. The phylogenetic tree was constructed using the neighbor joining method of the PHYLIP package.

Arabidopsis and rice were included to identify orthologs among the six Solanaceae species, rice, and Arabidopsis. Due to the higher sequence divergence of these two species, a lower number of orthologs can be expected, however, a total of 308 transcripts could be identified with reciprocal matches over all eight species. A phylogenetic tree was constructed based on the sequence alignment of the concatenated sequences from these 308 transcripts (see Figure 4). As these 308 transcripts are expected to be functionally conserved, their sequence divergence was used to assess the overall sequence divergence between the six Solanaceae species, Arabidopsis, and rice (Figure 4). Potato and tomato, as well as tobacco and *N. benthamiana *(both *Nicotiana *species), form closely related groups in the tree. Both petunia and the *Nicotiana *species are outliers among the Solanaceae, whereas pepper is more closely related to tomato and potato. As expected, Arabidopsis and rice form the outliers in the tree. These results further illustrate the process of sequence divergence during speciation of the Solanaceae.

### Identification of transcripts likely unique to Solanaceae

**Figure 5:**
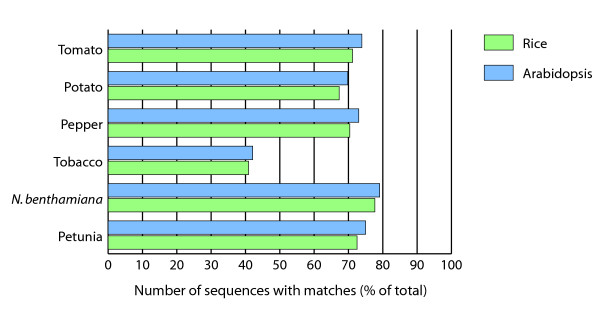
**Comparison of the six Solanaceae gene indices to Arabidopsis (blue) and rice (green). **Shown is the percentage of sequences of the Solanaceae gene indices with matches, BLAST score E value cut-off of 10^-5 ^in Arabidopsis and rice

Sequence information generated for a large number of plant species is primarily available in the form of EST collections while for Arabidopsis [22] and rice [23-25], near-complete genome sequences are available. To identify transcripts likely to be unique to the Solanaceae, the solanaceous transcripts were compared to 21 other gene indices as well as the predicted proteomes of rice and Arabidopsis to provide a representative sampling of plant genes. Like the Solanaceae, Arabidopsis is a eudicot whereas rice is a monocot. The Arabidopsis genome has been re-annotated since its completion [26] and the refinement of the annotation of rice is an ongoing process [27]. It is unlikely that a substantial number of novel new genes will be identified in either of these two species with the continuing annotation efforts, therefore comparison to these genomes is indicative of the number of Solanaceae transcripts not present in these two model species. From the comparison to the proteomes of Arabidopsis and rice (Figure 5), it appeared that there are a number of potentially novel or highly diverged transcripts among the Solanaceae family compared to Arabidopsis and rice. The percentage of sequences from potato, tomato, tobacco, *N. benthamiana *and petunia with significant matches (BLASTX using an E value cutoff of 10^-5^) in Arabidopsis varied between 70% (potato) and 79% (*N. benthamiana*). For rice, the percentages were slightly lower, between 67% (potato) and 78% (*N. benthamiana*), consistent with the eudicot nature of the Solanaceae. The sole exception to this high degree of conservation with these two model species is tobacco with only 42% of the tobacco gene index sequences matching an Arabidopsis protein and 41% matching a rice protein. As indicated in Figure 1, tobacco also has also a lower percentage of homologous sequences among other Solanaceae species examined in this study indicating the presence of unusual sequences within the available tobacco ESTs and ETs.

To further identify transcripts likely to be unique to the Solanaceae, all transcripts with no sequence similarity to Arabidopsis or rice were searched against 21 plant gene indices [28]. From the initial 116,207 transcripts, a total of 29,588 transcripts did not have any significant matches in Arabidopsis, rice, or the other 21 gene indices (see Table 4). With the exception of tobacco, the number of Solanaceae unique transcripts ranged between 15% for *N. benthamiana *and 22% for potato. The large number of transcripts without matches in these other plant species suggests that the Solanaceae contains unique sequences although this number may decrease as additional plant sequences become available in the future and more comparative analyses are performed. Overall the average length of these transcript assemblies was comparable to the overall average transcript assembly length; 420 bases compared to 531 bases for the singleton sequences, which were highly enriched in the Solanaceae-specific sequence data set. Transcripts with no matches in any of the 23 plant species or among Solanaceae are available in supplemental Table 2 [see Additional file 3].

**Table 4:** Identification of Solanaceae specific transcripts. Number of transcripts identified in the Solanaceae gene indices with no matches in Arabidopsis, rice or any of the 21 plant gene indices; * including Arabidopsis and rice.

	Total	Transcripts with no matches in Arabidopsis or rice	Transcripts with no matches in plant Gene Indices *
Potato	38239	10951 (29%)	8465 (22%)
Tomato	31838	7756 (24%)	6209 (20%)
Pepper	13003	3287 (25%)	2699 (21%)
Tobacco	21107	11671 (55%)	10251 (49%)
Petunia	4466	1064 (24%)	855 (19%)
*N. benthamiana*	7554	1481 (20%)	1109 (15%)

Next, we determined the number of transcripts unique to each of the six Solanaceae gene indices. Using TBLASTX, two different BLAST score cut-off E values were used to identify transcripts with no significant sequence homology within the Solanaceae. Using an E value cut-off of 10^-5^, 26% of the transcripts in any of the six Solanaceae gene indices had no match among the Solanaceae gene indices; using the more stringent E value cut-off of 10^-10^, 21% of the sequences had no match (see Table 5). Of these transcripts, 19% (E value cut-off of 10^-5^) or 16% (E value cut-off of 10^-10^) also did not have significant sequence homology in Arabidopsis, rice, or any of the 21 other plant gene indices; thus, these transcripts appear unique to each of the six Solanaceae gene indices based on these comparisons. The largest number of unique transcripts (38%) was found in tobacco in contrast to the 8–13% unique transcripts found in the other five solanaceous gene indices. These results indicate that in addition to a large number of conserved sequences among the Solanaceae, each species contained a subset of sequences likely to be unique to each species. As these transcripts also did not have significant homology to 21 other plant species for which sequence data is available, it is unlikely this can be attributed to differences in transcript sampling or the availability of a relatively low number of total sequences.

**Table 5:** Identification of Solanaceae species-specific transcripts. The left panel shows the number of sequences without matches in any of the Solanaceae gene indices. The right panel shows the number of sequences for each species without matches to Arabidopsis, rice, or any plant gene index, including Solanaceae.

	Unique among Solanaceae gene index		Solanaceae specific Transcripts	
	TBLASTX (10^-5^)	TBLASTX (10^-10^)	TBLASTX (10^-5^)	TBLASTX (10^-10^)
Potato	8878 (23%)	6967 (18%)	5796 (15%)	4825 (13%)
Tomato	6131 (19%)	4796 (15%)	3792 (12%)	3151 (10%)
Pepper	2483 (19%)	1921 (15%)	1865 (14%)	1531 (12%)
Tobacco	10580 (50%)	9131 (43%)	8949 (42%)	8076 (38%)
Petunia	825 (18%)	609 (14%)	631 (14%)	512 (11%)
*N. benthamiana*	1032 (14%)	757 (10%)	740 (10%)	571 (8%)

Total	29929 (26%)	24181 (21%)	21773 (19%)	18666 (16%)

### Expression profiling of solanaceous species

**Figure 6:**
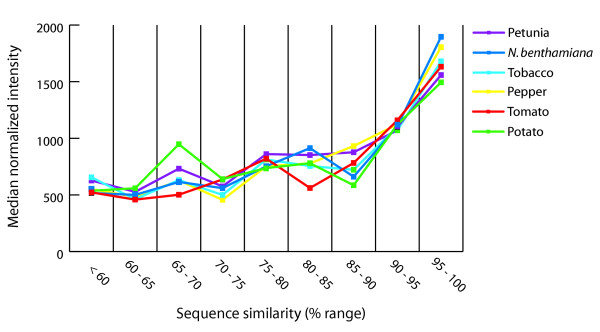
**Expression analysis of six solanaceous species. **Probes on the microarray were grouped according to the sequence similarity with potato and plotted against the median normalized signal intensity of each group. Shown is the average of two experiments of the median intensity of each group.

To experimentally validate the level of sequence conservation among the Solanaceae, global expression profiles in mature leaves were compared using microarrays. Potato microarrays containing ~12,000 potato cDNA clones were used to compare global gene expression patterns among the six Solanaceae species. As all probes on the microarray were derived from potato, we first assessed whether the potential sequence divergence of these probes would affect signal intensities. All probes on the potato array were searched against the other five Solanaceae gene indices and grouped based on BLASTN similarity score (5% bins) ranging from <60% to 95–100% sequence identity. Total RNA isolated from mature leaves of tomato, pepper, tobacco, petunia and *N. benthamiana *(query samples) was labeled with Cy3 and hybridized to the potato cDNA microarrays with RNA isolated from mature potato leaves that had been labeled with Cy5 (reference sample). The sequence similarity between the different Solanaceae species allowed for the detection of transcripts from the various species on the potato microarray for over 80% of the probes on the microarray. Normalized signal intensities were calculated for each element and the median intensity for each group of probes based on the BLASTN similarity score was plotted (Figure 6). Overall, the signal intensity increased for probes with a higher sequence similarity among the Solanaceae species, including potato. If this trend was attributable to the potential sequence divergence of the probes, it would be expected that the trend for potato would be different, as the potato RNA provides a perfect match to the probes on the array. Thus, the potential sequence divergence of the probes was not the limiting factor in reliable detection of expression levels for these heterologous hybridizations. This suggests that more highly conserved genes were expressed at relatively higher levels than more diverged genes because the group of probes with the higher sequence similarity all showed a higher median expression intensity. More conserved genes most likely represent "housekeeping" genes that can be expected to be generally expressed at higher levels. Alternatively, these probes may contain conserved motifs and therefore the probes on the microarray will cross-hybridize to multiple transcripts resulting in the higher signal intensities observed on the microarray for these elements. The number of clones that could be detected on the microarray was dependent on the species used as target. For the more diverged species, such as petunia, a lower number of clones were detected on the microarray (data not shown). Overall, we found similar expression levels in leaves across the six Solanaceae species used in this study (not shown), indicating that indeed sequence conservation may represent a functional similarity as well. In conclusion, the analyses of microarray data indicated that for the core genes conserved among Solanaceae with significant sequence similarity to potato, reliable gene expression values can be derived from microarrays with potato cDNA probes.

## Discussion

A high degree of sequence conservation among Solanaceae family members had been suggested previously based on small scale assays and analysis. Here, we report for the first time, a large scale comparison of six Solanaceae family members. Although the analyses in this study confirmed the high degree of sequence conservation, they also revealed a large number of Solanaceae specific transcripts and sequence divergence among Solanaceae.

### Transcript sampling for the Solanaceae gene indices

To date, only a limited amount of genomic sequence data is (publicly) available for the Solanaceae. Therefore, the EST sequence data assembled in this study was used to assess the diversity of transcripts among the Solanaceae. The assessment of the annotation by GO terms of the six gene indices indicated an overall similar functional composition of the transcripts. In addition, the analysis of GC content was consistent with Arabidopsis and among the Solanaceae, with tobacco being the exception. These data show that the sequences used in this study provide a valid representation of the various solanaceous genomes. The wide range of different library sources of the sequences did not affect the number of sequence matches among the different Solanaceae species, indicating the absence of a high percentage of tissue specific transcripts. This can be explained by the close developmental relationship between most plant organs as flowers can be considered modified leaves and stolons as modified stems. A low number of tissue specific transcripts were also observed in Arabidopsis using Massive Parallel Signature Sequencing [29]. Among five different libraries of callus, inflorescence, leaves, roots and siliques, less than 0.25% of the transcripts showed tissue specificity [29]. Also in maize, using cDNA microarrays, only 7% of the genes were expressed in a highly tissue specific manner among seven different organs of maize [30]. In contrast, the assessment of the frequency of the EST sequences can be used for the comparative analysis to evaluate differential expression. This approach has been used for tomato and potato [16,31], but can only be successfully employed with a large number of diverse libraries and deep sequencing as most tissue specific transcripts may be expressed at low levels and therefore be relatively rare and not be sampled by sequencing.

A single microarray platform was successfully applied for heterologous hybridization of Solanaceae species. For transcripts with significant sequence similarity to the potato probes on the cDNA microarray, reliable expression data could be obtained. Similar hybridization characteristics were found using heterologous hybridization to a fish cDNA microarray [32]; the number of elements that could be detected on the microarray was correlated with the phylogenetic distance. Cross-species hybridization was also shown for human and bovine orthologous genes on a human cDNA microarray [33]. The global expression data indicated that the conserved transcripts were expressed similarly among leaf tissue of the six Solanaceae species examined.

### Solanaceae species contain unique transcripts

Overall, a high degree of sequence conservation among the Solanaceae was observed in accordance with previous small scale studies [6]; for up to 81% of the gene index sequences, significant matches at the nucleotide level could be found within the Solanaceae, consistent with the level of sequence conservation observed at the protein level. Using a more stringent approach of orthology revealed that for the largest gene indices of potato and tomato, a putative ortholog could be identified at the nucleotide level for 47% of the unique transcripts in the gene index. In addition, comparison of the Solanaceae gene indices to Arabidopsis, rice, and 21 other gene indices revealed transcripts without matches to these non-solanaceous species as well as transcripts without matches to individual Solanaceae species. Depending on the stringency of alignment, 16–19% of the transcripts did not have a match among the plant sequences examined. A similar approach was used to identify transcripts specific for legumes [34]. These results show that between these closely related species there was still substantial sequence divergence, which was supported by the sequence divergence among 308 orthologous transcripts of six Solanaceae, Arabidopsis and rice. The available EST sequences only provide a snapshot of the genome, thus the number of unique transcripts may be lower but still be substantial as the transcript sampling among the Solanaceae proved to be a representative sampling. The large number of EST sequences available for tomato and potato were likely to contain the most abundant transcripts, so a large number of transcripts without sequence homology is likely to remain with increased EST sequencing until more sequence data is generated.

The outlier for most analyses appeared to be tobacco with a low number of significant matches among Solanaceae, Arabidopsis, and rice. No obvious explanation could be found for this but it is unlikely that tobacco will contain a much higher plant specific gene content. Matsuoka et al. [35] report on the EST sequencing of a cell suspension library of tobacco, which was the origin of a large portion of the tobacco gene index. In this study, a low number of tobacco sequences matched sequences from other plant species, consistent with our analyses. The GO assignments and the identification of orthologs indicated that the tobacco sequence sample did contain similar transcripts as the other five Solanaceae gene indices, validating the general conclusions for the Solanaceae species in this study, including tobacco.

The finding of a large number of transcripts without matches among the Solanaceae species will complicate the efforts of establishing a single reference genome for the Solanaceae by sequencing a single representative species. Although a large level of synteny exists between the Solanaceae, it is unclear how novel genes evolved and whether there is a large difference in gene content among the Solanaceae. Fortunately, for three Solanaceae species (tomato, potato and tobacco), genome sequencing projects are in progress. The availability of three draft genome sequences will allow for the detailed analysis of genome conservation and understanding of the genes involved in the different phenotypes within the Solanaceae.

## Conclusion

In summary, this study documents for the first time the genomic scale comparison of the available coding sequences (ESTs and ETs) from six Solanaceae species. Sequence comparisons at the nucleotide level among potato, tomato, pepper, eggplant, tobacco and *N. benthamiana*, including ortholog analysis, confirmed a high level of sequence conservation. In addition, phylogenetic analysis and comparative analyses with Arabidopsis, rice and 21 other gene indices revealed sequence divergence during speciation as evidenced by transcripts likely unique among the Solanaceae and unique to individual Solanaceae species. Global expression profiling showed similar expression patterns of conserved genes in mature leaves among the six solanaceous species.

## Methods

### Computational methods

Gene indices were constructed essentially as described [15]. In summary, all available sequences for potato, tomato, pepper, eggplant and petunia were collected from Genbank and sequences with over 94% sequence identity over 40 or more bases with unmatched overhangs of 30 bases in length were placed in clusters using the Paracel Transcript Assembler to generate tentative consensus sequences (TC) and singleton ESTs and ETs. The TCs were searched against a non-redundant protein database to provide a putative annotation for the TC, with a minimum of 30% identity over 20% of the length of the translated TC. All gene indices are available at [28]. The 21 gene indices used for searches against the Solanaceae gene indices were: Ice plant (v4.0), Cocao (v1.0), Cotton (v6.0), Grape (v4.0), Barley (v9.0), Sugar beet (v1.0), *Brassica napus *(v1.0), Sunflower (v3.0), Lettuce (v2.0), Lotus (v3.0), Wheat (v10.0), Maize (v15.0), *Medicago truncatula *(v8.0), Onion (v1.0), *Pinus *(v5.0), Poplar (v2.0), Rye (v3.0), *Sorghum bicolor *(v8.0), Sugarcane (v2.1), Soybean (v12.0) and Spruce (v1.0). GO terms were transitively annotated based on sequence similarity (E value cutoff of 10^-10^) to Arabidopsis proteins (Release 5, [26] which has been manually curated for molecular function GO terms. The Plant/GOSlim reduced ontologies were used [36].

Each of the six gene indices was pair-wise matched against the other gene indices using WU-BLAST [37] with BLASTN and TBLASTX options. BLAST scores were filtered for significant hits using an E value cut-off as indicated in the text. Each of the six gene indices were searched against the predicted rice and Arabidopsis proteome using BLASTX and the top hit was picked for each entry of the gene indices using an E value cutoff of 10^-5^. Putative orthologs among the six Solanaceae species, rice and Arabidopsis were identified essentially as described [21]. In summary, the non-redundant sets of eight gene indices were compiled and searched against each other using BLASTN. The reciprocal best hit pairs with a cutoff E value 10^-10 ^were clustered to generate the ortholog groups. 308 clusters which contain at least one transcript from each of the 8 species were selected and one representative sequence for each species was chosen for each group by counting the reciprocal matches in the clusters. Multiple sequence alignments for each of the 308 clusters were performed and sequences in both ends without consensus matches were removed. Sequences from each species were concatenated together in the same order and aligned to each other using CLUSTAL W [38]. A neighbor joining tree was generated using PHYLIP (Phylogeny Inference Package) (Felsenstein, J. 2004, distributed by the author. Department of Genome Sciences, University of Washington, Seattle).

### Microarray hybridizations and data analysis

Potato cDNA microarrays were constructed as described [39]. Potato, tobacco, tomato, petunia, pepper and *N. benthamiana *plants were grown in Percival growth chambers (Percival Scientific, Inc. Perry, IA) at 25°C and 16 h light for 4–6 weeks. Total RNA was extracted from mature leaves using the Qiagen RNAesy kit (Qiagen, Valencia, CA) and labeled as described previously [39]. Hybridization and washing was performed essentially as described [39]. After the final washing step and spin-drying of the slide, slides were scanned using an Axon scanner at maximum laser power (Axon Instruments, Union City, CA) at both 532 and 635 nm. The PMT values for both wavelengths were adjusted to capture a similar number of normalized counts for each channel.

The TIFF images were quantified using Genepix 5.0 (Axon Instruments, Union City, CA). The software automatically flags spots that cannot be found in one of the channels; these are flagged and excluded from further analysis. Spots containing > 30% saturated pixels in either channel or a diameter <70 μm were flagged and not used for subsequent analysis. Local background was subtracted from the signal value (mean pixel intensity). The data were normalized using the quantile method in the limma package [40] of BioConductor [41]. Flagged spots were given a weight of 0 using the weight function within the package which excludes these spots from affecting the normalization. All analyses used the average of the two on-slide replicates. If one of the two replicates was flagged, the remaining value was used for analysis.

The microarray data are available at the TIGR Potato functional genomics and Solanaceae resources web pages [42] and through the Gene Expression Omnibus (GEO) [43] under platform accession GPL1901.

## Authors' contributions

WAR coordinated and designed the study, performed the microarray data analysis and drafted the manuscript. DL constructed the Gene Indices, performed the analysis of orthologs, GC content and constructed the phylogenetic tree. JL performed all the BLAST searches and analyses. SI carried out the microarray hybridizations. SO performed the assignment of GO terms. CRB performed the library composition analysis of the Gene Indices, participated in coordination and design of the study and helped to draft the manuscript. All authors read and approved the final manuscript.

## Supplementary Material

Additional File 1 Supplemental Table 1 (split into two files) containing the Solanaceae ortholog clusters, for each cluster the TC numbers from each gene index are listed that form an ortholog cluster. Click here for file File 2

Additional File 3 Supplemental Table 2 containing the Solanaceae specific transcripts, for each of the six solanaceous species the TC numbers are listed without matches in 23 other plant species (see Table 4), transcripts unique to Solanaceae (Table 5, right panel, TBLASTX E-05) and transcripts unique to each species (Table 5, left panel, TBLASTX E-05). Click here for file
